# Alpha-melanocyte stimulating hormone (α-MSH): biology, clinical relevance and implication in melanoma

**DOI:** 10.1186/s12967-023-04405-y

**Published:** 2023-08-22

**Authors:** Luigi Dall’Olmo, Nicole Papa, Nicoletta Concetta Surdo, Ilaria Marigo, Simone Mocellin

**Affiliations:** 1 Department of Surgical Oncological and Gastroenterological Sciences, Padua University, Via Giustiniani 2, 35128 Padua, Italy; 2 Istituto Oncologico Veneto IOV-IRCCS, 35128 Padua, Italy; 3 Neuroscience Institute, National Research Council of Italy (CNR), 35121 Padua, Italy; 4 Veneto Institute of Molecular Medicine VIMM, Foundation for Advanced Biomedical Research, 35129 Padua, Italy

**Keywords:** Melanoma, α-MSH, MC1R, Melanoma resistance, Anticancer strategies

## Abstract

Alpha-melanocyte stimulating hormone (α-MSH) and its receptor, melanocortin 1 receptor (MC1R), have been proposed as potential target for anti-cancer strategies in melanoma research, due to their tissue specific expression and involvement in melanocyte homeostasis. However, their role in prevention and treatment of melanoma is still debated and controversial. Although a large body of evidence supports α-MSH in preventing melanoma development, some preclinical findings suggest that the α-MSH downstream signalling may promote immune escape and cancer resistance to therapy. Additionally, in metastatic melanoma both MC1R and α-MSH have been reported to be overexpressed at levels much higher than normal cells. Furthermore, targeted therapy (e.g. BRAF inhibition in BRAF^V600E^ mutant tumours) has been shown to enhance this phenomenon. Collectively, these data suggest that targeting MC1R could serve as an approach in the treatment of metastatic melanoma. In this review, we explore the molecular biology of α-MSH with particular emphasis into its tumor-related properties, whilst elaborating the experimental evidence currently available regarding the interplay between α-MSH/MC1R axis, melanoma and antitumor strategies.

## Introduction

Melanocortins are peptidic pituitary hormones produced by the cleavage and posttranslational modifications of pro-opiomelanocortin hormone (POMC). The family of melanocortins includes Adrenocorticotropic Hormone (ACTH), Melanocyte Stimulating Hormone (MSH) and endorphins, that activate five forms of membrane receptors called Melanocortin Receptors (MCRs) with different affinities**.** MSH consists of the three forms α-, β- and γ-MSH. Among them α-MSH is well-characterized and first described for its melanin-inducing activity in frogs. α-MSH is a 13 amino acid neuropeptide secreted by melanocytes and keratinocytes after ultraviolet light exposure and it is responsible of the melanin synthesis, being the main actor of skin pigmentation [1–3]. Moreover, it has been shown that α-MSH and analogues have anti-inflammatory and anti-microbial properties, activating melanocortin receptors (MCR) signaling [4, 5]. α-MSH binds to four out of five MCR subtypes (MC1R, MC3R, MC4R, MC5R), regulating several downstream cascades in different cell types. Notably, in melanocytes MC1R is highly expressed and the binding of α-MSH promotes the expression of melanogenesis enzyme genes via Adenylyl Cyclase (AC)/cyclic AMP (cAMP)/Protein Kinase A (PKA) pathway. Beyond melanin synthesis, the α-MSH/MC1R axis controls a plethora of important processes such as DNA damage repair, reduction of free radical production and cell proliferation among others. For the broad spectrum of properties, the use of α-MSH or its synthetic analogs has been proposed for several pathologic conditions. The primary target cell for α-MSH is the melanocyte, in which, despite the proven efficacy in the prevention of melanoma development, its role in malignant melanoma, and in particular in metastatic stage disease still remains underinvestigated [6].

## 2- Molecular biology of α-MSH

### α-MSH production and melanocortin receptors

Human POMC gene is located on chromosome 2p23.3 and it is expressed in a variety of tissues but broadly in testis, pancreas and fat tissue. The early encoded protein undergoes extensive posttranslational processing via prohormone convertases cleavage, in order to produce at least ten active peptides mainly synthesized in corticotroph cells of the anterior pituitary. Among them, ACTH is essential for physiologic steroidogenesis whereas in other tissues such as placenta and epithelium, proteolytic cleavage gives rise to peptides with roles in energy homeostasis, melanocyte stimulation, and immune modulation. These include several distinct melanotropins (or melanocortins): α-, β- and γ-MSH. All forms of MSHs bind to four well characterised G-Protein Coupled Receptor (GPCR) subtypes: Melanocortin Receptors (MC1R, MC3R, MC4R, and MC5R), whereas MC2R is specific for the binding with ACTH [7–10].

MC1R is an intron less gene encoding seven pass transmembrane GPCR, preferentially expressed on cell membrane of melanocytes and mainly recognized as the key regulator of the synthesis of epidermal melanin pigments [11, 12]. MC1R gene is polymorphic and frequent variants are associated not only with hair/skin phenotypes but also with increased melanoma risk [13–16]. MC1R is also the target of the α-MSH antagonists Agouti protein and Agouti related protein (Agrp), both responsible for the inhibition of eumelanin production in favour of pheomelanin [17].

MC3R and MC4R genes encode the GPCRs for MSH and ACTH and are expressed in tissues other than the adrenal cortex and melanocytes. Studies suggest a function role of MC3R and MC4R in the regulation of energy homeostasis and food intake. Mutations of this receptors have been correlated to susceptibility to obesity and anorexia in humans [18–22]. Evidence suggests that MC5R plays a key role in the regulation of sexual behaviour, thermoregulation and exocrine secretion (sebogenesis) but also in immune reaction and inflammatory response via cAMP signal transduction [23, 24].

### α-MSH regulation of melanocyte function: MC1R/cAMP signaling cascade

MC1R plays a key role in cutaneous homeostasis and photoprotection as it is coupled to the stimulatory G protein Gα which in turn activates AC switching on the cAMP/PKA pathway [25].

**Fig. 1:**
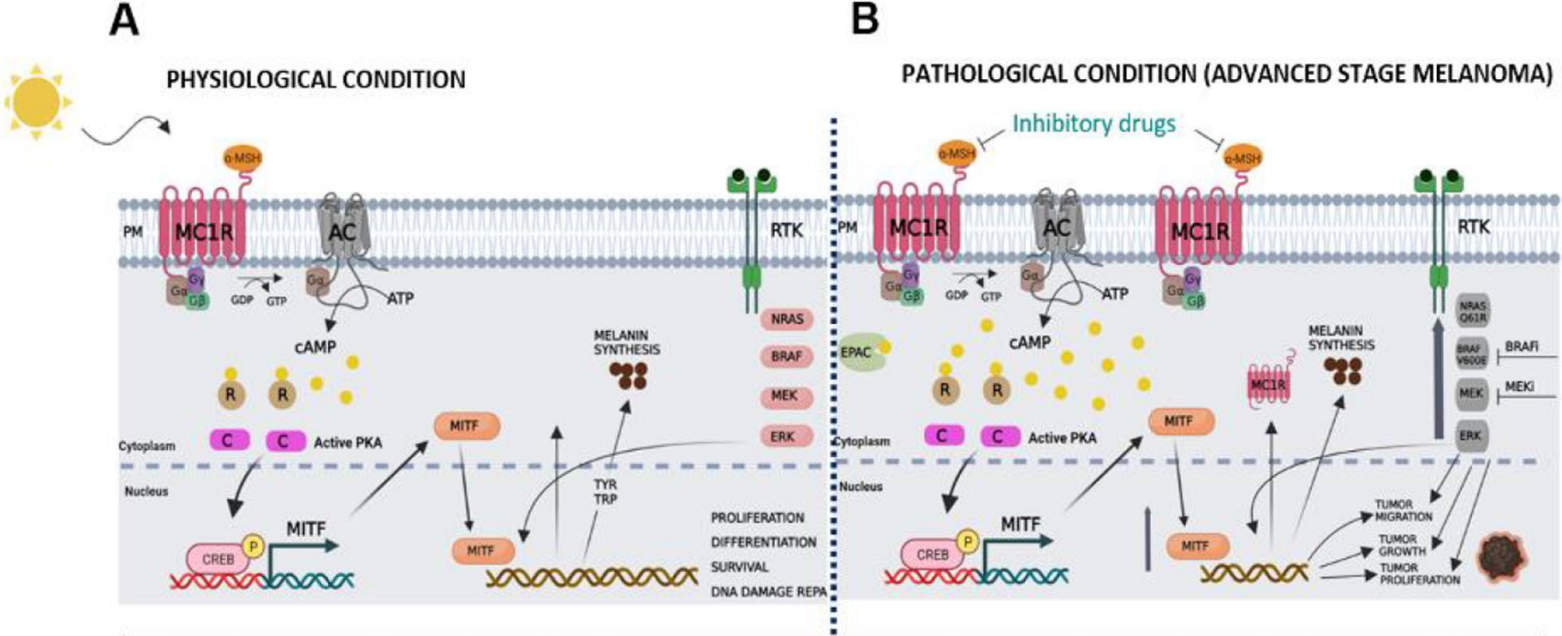
**A** Physiological condition. **B** Pathological condition (advanced stage melanoma). In physiological condition melanocytes express a membrane receptor (MC1R) that controls the melanin synthesis process. **A** Upon UV exposure, alpha-melanocyte stimulating hormone (α-MSH) is released by keratinocytes: the binding of α-MSH to MC1R activates Adenyl Ciclase (AC) that stimulates cilic AMP (cAMP) production and the activation of Protein Kinase A (PKA). PKA phosphorylates the transcription factor CREB that stimulates the transcription factor MiTF which in turn promotes the expression of melanogenesis enzyme genes TYR, TRP1 and DCT. In our working hypothesis **B** in advanced stage of melanoma, tumour cells overexpress MC1R and BRAF inhibitor treatment significantly increase this MC1R expression via MiTF-dependent pathways, leading to enhanced ligand binding on the cell surface. As a consequence, the cAMP/PKA pathway is aberrantly altered and might promote tumour migration , growth and proliferation. PM: Plasmatic Membrane; Gα, Gβ, Gγ: G proteins; CREB: cAMP Response Element Binding protein; RTK Tyrosine Kinase Receptor. This figure was created with www.BioRender.com

PKA phosphorylates the transcription factor cAMP Response Element Binding Protein (CREB) that stimulates the Microphthalmia inducing Transcription Factor (MiTF) which in turn promotes the expression of melanogenesis enzyme genes Tyrosinase (TYR), Tyrosinase Related Protein 1 and 2 (TRP1,TRP2) and Dopachrome Tautomerase (DCT) [26, 27]. MiTF coordinates a broad range of biological processes including cell survival, differentiation, proliferation, migration, invasion, senescence, metabolism, and DNA damage repair (Fig. 1).

α-MSH stimulated MC1R triggers the production of both free radicals (ROS) and brown/black eumelanin, acting as a filter against UV. MC1R polymorphisms are associated with pigmentary phenotypes such as Red-Hair-Colour (RHC) and light skin [28, 29]. Patients carrying these variants show a reduced ability to produce eumelanin and therefore pheomelanin synthesis prevails. Pheomelanin acts as a photosensitizer and these patients are more susceptible to skin cancer development, both by UV- dependent and independent mechanisms [30].

### Other pathways connected with α-MSH/MC1R signal

The Mitogen-Activated Protein Kinase (MAPK) signal transduction cascades are highly conserved regulators of cell proliferation, differentiation and survival which are activated by signals as cytokines, growth factors and other stress inducers. The most widely studied MAPK pathway is the RAS/RAF/MEK/ERK cascade that controls melanogenesis and it is aberrantly activated in 90% of human cutaneous melanomas as well as in several type of cancers. Gain of Function (GoF) mutations in N-RAS and B-RAF are common drivers for melanoma development (~ 25% for N-RAS and ~ 60% for B-RAF) as they are responsible for dysregulated cell cycle and proliferation [31–35]. Multiple stimuli such as growth factors, cytokines, viruses, GPCR ligands and oncogenes can sequentially activate the ERK pathway and result in ERK1/2 phosphorylation that regulates different transcription factors, including c-FOS, cJUN, ELK-1, c-MYC, and ATF-2 controlling cell growth, migration, and differentiation [36]. Noteworthy, ERK1/2 can phosphorylate MiTF decreasing its protein levels and leading to a negative regulation of melanogenic enzymes, inhibiting melanogenesis process. In human melanocytic cells ERK activation upon α-MSH binding to MC1R is a cAMP-independent process, it occurs through a transactivation mechanism of the Tyrosine Kinase Receptor (RTK) c-KIT and plays an important role in melanogenesis [37–39].

Another pathway linked to α-MSH/MC1R axis is PI3K/AKT, an intracellular signal transduction cascade that, through the phosphorylation of several downstream substrates, is involved in cellular functions such as cell growth, proliferation, and differentiation. The key molecules involved in this signalling pathway are RTKs, phosphatidylinositol 3-kinase (PI3K), phosphatidylinositol-4,5-bisphosphate (PIP2), phosphatidylinositol-3,4,5-triphosphate (PIP3) and AKT/protein kinase B. The binding of RTK with growth factors and various stimuli activates PI3K which in turn phosphorylates PIP2 leading to the production of the second messenger PIP3 that regulates metabolic processes by recruiting signaling proteins, including AKT/Protein kinase B (PKB) [40]. PTEN (Phosphatase and TENsin homolog) is a phosphatase responsible for the conversion of PIP3 to PIP2, acting as an antagonist of the PI3K/AKT response. Investigating the interaction between MC1R and PI3K/PTEN signaling, it has been shown that upon α-MSH binding, MC1R interacts with PTEN and, by preventing its degradation, inactivates AKT. It has also been shown that RHC MC1R allelic variants have an impaired ability to interact with PTEN, thus increasing AKT signaling and predisposing melanocytes to melanomagenesis [41]. Studies with a synthetic analog of α-MSH revealed that the stimulation of RHC MC1R variants activates DNA repair pathways through a cAMP-independent mechanism mediated by AKT activation [42]. On the other hand, it has been shown that the binding of α-MSH to MC1R activates DNA repair and antioxidant signals in a cAMP-dependent manner with decreased AKT phosphorylation [43]. Moreover an interplay between α-MSH/MC1R and Peroxisome Proliferator-Activated Receptor-γ (PPAR-γ) has been reported [44]. Briefly, α-MSH induces the release of calcium (Ca^2+^) from endoplasmic reticulum (ER) by a phospholipase C (PLC) dependent mechanism and Ca^2+^ efflux is connected with the translocation of PPARγ into the nucleus, where it promotes the transcription of target genes involved in lipid metabolism, adipogenesis, maintenance of metabolic homeostasis, inflammation and anticancer effects in a variety of human tumours [45].

## α-MSH/MC1R: range of action

### Maintenance of cell integrity and DNA damage repair. MC1R polymorphism

In physiologic conditions, the main role of α-MSH is to protect skin from UV exposure by coordinating the production of eumelanin. However, both in melanocytes and keratinocytes, several studies have established that the α-MSH/MC1R-cAMP axis is also involved in additional responses, like antioxidant defences and DNA damage repair [42, 46]. UV radiation and melanin synthesis process are sources of ROS among which hydrogen peroxide (H_2_O_2_), that is able to injure all cell compartments [47]. After UV exposure, human melanocytes stimulate the generation of H_2_O_2_ with a concomitant decrease in the activity of catalase, the enzyme most involved in H_2_O_2_ neutralization [48]. Therefore, it has been shown that treatment with α-MSH protects melanocytes from oxidative stress since α-MSH through MC1R induces both the activation and overexpression of catalase, reducing H_2_O_2_ production [49, 50].

Exposure to UV radiation is considered the most common environmental risk factor for skin melanoma. The high prevalence of polymorphisms of MC1R, with more than 300 variants, makes it the best-established susceptibility gene for cutaneous melanoma [25, 51]. The association between some MC1R polymorphisms and red hair, freckles, and inability to tan (the RHC phenotype) was first reported in 1995 by Valverde et al*.* [52]. An extensive body of research shows that inactivating variants of MC1R are the main contributors to the increased risk of melanoma development, because the functions of UV protection and DNA damage repair are lost. According to their penetrance RHC MC1R alleles have been classified as high (R) or low (r) variants. “R” variants include D84E, R142H, R151C, R160W, and D294H and people carrying these variants MC1R have the highest risk of developing melanoma and non-melanoma skin cancers whereas “r” variants: V60L, V92M, and R163Q showed a weaker association with the RHC phenotype [52–56].

In keratinocytes, the canonical α-MSH/MC1R-cAMP-PKA pathway enhances Nucleotide Excision Repair (NER) activity: PKA directly phosphorylates the DNA damage sensors Ataxia Telangiectasia Mutated (ATM) and Rad3 related (ATR) which actively recruits the key NER protein Xeroderma Pigmentosum complementation group A (XPA) to sites of nuclear UV damage, thus accelerating the clearance of UV-induced lesions and reducing the mutagenesis rate [57].

It has been reported that α-MSH-MC1R axis can induce cutaneous carcinogenesis other than melanoma. Regarding Non-Melanoma Skin Cancers (NMSCs), it must be highlighted that carriers of two MC1R variant alleles have a higher risk of developing NMSC than the WT. However, it is not clear whether MC1R variants confer a relevant contribution in the genesis of skin carcinomas [58].

### Anti inflammatory and immunomodulatory properties

In addition to its effects on melanocytes, α-MSH has potent anti-inflammatory effects when administered systemically or locally [59]. Its immunomodulating properties rely mainly on the binding with MC1R that is also expressed on monocytes, macrophages, and dendritic cells (DCs). α-MSH downregulates the production of pro-inflammatory cytokines IL-1, IL-6, TNF-α, IL-2, IFN-γ, IL-4, IL-13 and in contrast, anti-inflammatory IL-10 production is upregulated. At the molecular level, α-MSH affects several pathways implicated in the regulation of transcription factors such as NFκB thus modulating inflammatory cell proliferation, activity and migration. NFκB regulates the transcription of genes involved in cell survival, and inhibition of NFκB activation has been considered as a strategy for the treatment of melanoma [60–63]. α-MSH was discovered to be an ancient natural antimicrobial agent against two representative pathogens Staphylococcus A. and Candida A., enhancing the local inflammatory reaction. It has been described that the candidacidal activity is mostly based on increasing intracellular cAMP levels that interferes with microbial regulatory pathway thus reducing fungal viability and germ tube formation [64].

From an oncological perspective, in human melanoma cells, an anti-inflammatory and anti-invasive effects of α-MSH have been reported [65].

### Broad spectrum of α-MSH applications

The pivotal role of α-MSH in stimulating skin pigmentation and protecting from UV damage led to propose its topical application as strategy to improve a “sunless tanning” both for cosmetic purpose and mostly as skin cancer prevention. Therefore, by boosting the α-MSH/MC1R-cAMP/PKA pathway activation and MiTF transcription, melanogenesis and DNA damage repair apparatus are enhanced [66].

Moreover, studies revealed that α-MSH and synthetic analog peptides could be resolutive for other conditions as Hypoactive Sexual Desire Disorders (HSDD) or be neuroprotective against cerebral ischemia/reperfusion injury as well as neovascularization inhibition [67–69]. Additionally α-MSH was found to be involved in appetite regulation (suppressor), in the pathogenesis of restless legs syndrome and in insulin resistance/sensitivity [70–73].

## α-MSH/MC1R and cancer

### Melanoma

Cutaneous malignant melanoma arises from melanocytes, the pigment producing cells, and remains a challenging disease due both to difficult early diagnoses and to the tendency to metastasize quickly to lymph nodes and distant organs such as liver, lung and brain. Although melanoma accounts for only about 10% of skin cancers it is responsible for the vast majority of deaths [74, 75].

Mortality is correlated with the stage at diagnosis and, to date, the management of metastatic disease remains a relevant clinical issue. Genetic mutations in oncogenes and tumour suppressor genes affecting the RAS-RAF-MEK-ERK signalling pathway (MAPK) are the main drivers in most cutaneous melanomas. A common mutation found in melanoma patients is BRAF^V600E^ whereas tumours bearing NRAS mutations are less frequent but more aggressive and associated with shorter survival [76]. The MAPK cascade leads to activation of ERK1 and ERK2 which translocate into the nucleus to regulate MiTF, cMYC and other transcription factors to sustain cell cycle progression, tumor invasion, metastasis and immune evasion [77].

The BRAF^V600E^ mutation is found only in about 50% of melanoma and this fact limits the use of BRAF inhibitors (BRAFi). Moreover, most of patients in BRAFi therapy for metastatic melanoma relapses early after an initial partial response. The development of drug resistance within some metastatic clones causes the relapse of disease.

### MC1R overexpression in melanoma

α-MSH/MC1R/cAMP axis converges to the regulation of MiTF expression with a pivotal role for homeostasis but when impaired in melanoma environment it takes a role in tumor progression and survival. It has been reported that MiTF is a factor that supports melanoma stem cells properties [27, 78, 79].

Many studies showed increased levels of MC1R expression on the surface of most melanomas (either primary or metastatic tissues) but not in carcinoma cell lines making it a valuable marker of melanoma cells [80, 81]. Moreover, the tumor itself overproduces α-MSH, leading to an autocrine hyperproliferative process, described in melanoma metastases [82].

### EPAC in melanoma

cAMP regulates a wide range of physiologic processes in melanocyte homeostasis mainly by acting through the canonical PKA-CREB pathway. During melanoma initiation the system might switch and impaired cAMP signaling might sustain the tumor environment in a way that need to be explored deeply. However Rodriguez et al*.* showed that topical application of forskolin that directly activates AC, increases the level of cAMP, speeding melanoma tumor development in BRAF^V600E^/PTEN mouse model of melanoma and stimulating the proliferation of mouse and human primary melanoma cells in vitro. Although the process was cAMP-driven, an alternative downstream effector called Exchange Protein directly Activated by cAMP (EPAC) is involved. EPAC has been identified in 1998 and it acts as a guanine nucleotide exchange factor for the GTPase Ras family: RAP1 and RAP2 [83]. Modulating different signaling pathways, EPAC is involved in several cellular processes such as cell proliferation, migration, apoptosis and adhesion in various tissues [84]. In addition it has been shown that MC1R-cAMP-EPAC cascade promotes DNA repair by increasing the nuclear translocation of XPA protein in keratinocytes [57]. On the other hand, EPAC has shown to have a pro-metastatic role as it acts by activating ERK pathway and α_v_β_3_ integrin through RAP1 thus promoting tumorigenesis and migration in human lung cancer cells but also by influencing other signalling cascades in cells derived from human metastatic melanomas, in human melanoma samples and melanoma cell lines [85–87]. The current hypothesis is that EPAC could have a different function during different stages of melanoma progression, with EPAC-RAP1 axis showing both a pro-survival role in primary melanoma and an anti-survival role in metastatic melanoma. Hence, it could be speculated that proliferation is inhibited during metastasis promoting an invasive phenotype [88, 89].

## α-MSH-based strategies in melanoma treatment

The MC1R receptor is recognized to play a key role in melanocyte, melanosome, and melanoma cell (patho)physiology. Regarding metastasis, overexpression levels of MC1R, and MSH production by the neoplastic tissue itself are well-established data in the scientific literature. In this way, metastasis creates and self-maintains an autocrine loop that stimulates the growth, proliferation and invasiveness of the neoplasm, with the possibility of recurrence at metastatic sites, progression and dissemination, creating new metastatic sites and thus making the patient life-threatening.

This mechanism may also play a role in resistance to targeted therapy against mutated B-RAF (V600E B-RAF) where this phenomenon is described to be enhanced, suggesting that MC1R activation may contribute to the development of cancer resistance to dabrafenib. For these reasons, our group among others posits MC1R inhibition as a possible strategy to counteract this autocrine loop that intervenes in metastatic disease.

MC1R potentially constitutes an ideal target for design of novel anticancer drugs both for its involvement in melanocytic pathophysiology anf for its high levels of tissue-specific expression in melanoma cells.

At present, many works have shown promising results using the tissue specificity of MC1R for melanocytic tissues as an antitumor strategy.

Liu and collaborators reported that the immunotoxin α-MSH-PE38KDEL, constructed by connecting the α-MSH gene to PE38KDEL (a mutated and truncated form of a bacterial toxin), showed in vitro high cytotoxicity on MC1R positive melanoma cell lines, promoting apoptosis via Erk1/2/MITF/TYR signaling modulation in a MC1R-dependent manner [90]. They demonstrated that MC1R is essential for the immunotoxin-mediated cytotoxicity, promoting melanoma cell apoptosis inhibiting MITF and TYR expression. In fact, the overexpression of MITF or TYR abolishes α-MSH-PE38KDEL induction of apoptosis in mouse melanoma B16-F10 cell line. The authors demonstrated that the same pathway modulation significantly inhibited the in vivo tumor-forming ability of B16-F10 cells, when injected into athymic BALB-C nude mice.

Other works, using radionuclide-αMSH analogs conjugates, depicted interesting results in a theranostic settingThese studies, conducted in melanoma-bearing mouse models, demonstrated the high specificity of those molecules for MC1R, with a good bioavailability and renal clearance. In in vivo preclinical experimental animal model bearing mouse B16F1 or B16F10 melanoma radiolabelled peptides targeting MC1R, the radionuclide-αMSH analogs conjugates are able to selectively and specifically kill melanoma cells, sparing healthy cells and normal tissue. These studies are reviewed and summarised in two recent works [91, 92] Shi and collaborators considered studies about molecular probes for melanoma theranostics targeting either MC1R or melanine. These MC1R targeted radiotracers, displaying a good tumor uptake and retention, could potentially be used for inmaging of MC1R expressing melanoma in clinic. These imaging probes could be transformed into therapeutic radiopharmaceuticals through radiolabeling with beta- or alpha emitters. [91].

These novel sensitive and specific MC1R targeted radiotracers can overcome the actual limitation of (18)F FDG PET (I.e poor selectivity for distinguishing tumor from inflammatory tissue and low sensitivity in the detection of both nodal and lung and brain metastases) [92]. Furthermore, in a potential clinical application, cytotoxic radiation generated by therapeutic radionuclides could help treat remnant metastatic deposits, in an adjuvant setting, after surgical excision of the tumor.

Notheworthely, the group of Cachin reported the results of a multicenter phase III clinical trial [93]. This trial evaluated the accuracy of a new benzamide-derivative melanin targeted radiotracer, the (123)I-BZA2 radiopharmaceutical. This trial was prematurely closed after the enrollment of 87 patients, because of the low sensitivity of the radioconjugate in comparison to (18)F FDG when considering both a patient-based and a lesion-based analyses. However, (123)I-BZA2 demonstrated higher specificity than (18)F FDG for diagnosis of melanoma metastasis in a lesion-based analysis.

Further clinical studies are needed to validate the results of promising pre-clinical works.

## Conclusions and perspectives

In this study we reviewed and summarised the molecular biology of α-MSH/MC1R, their range of action beyond pigmentation, the role of α-MSH/MC1R axis in melanoma and the MC1R targeting therapeutic strategies that have been proposed for melanoma.

α-MSH is the key hormone for melanocytic metabolism. It is not only the main actor of skin pigmentation but it displays also anti-inflammatory and anti-microbal properties. Among melanocortin receptors, melanocytes mainly express MC1R, whose binding with α-MSH promotes both the production of eumelanin, through the activation of AC/cAMP/PKA pathway, and melanocytic proliferation, survival and migration.

In summary, this review shows the ambivalence in the relationship between α-MSH and its membrane receptor. In physiological condition, the intracellular pathways elicited by this bond ensure skin pigmentation, DNA repair and anti-microbal and inflammatory defense. On the other hand, in pathological conditions, the overstimulation of the α-MSH/MC1R axis can lead to survival, uncontrolled proliferation, and invasion of cancer cells in metastatic melanoma. Moreover, some reports showed that synthetic alpha-MSH analogues (MC1R agonists) could lead to proliferation of melanocytic cells in predisposed patients, representing an increased risk for atypical naevi and melanoma development [94, 95]. Notheworthely, Kansal et al., reported that the inhibition of MC1R diminishes melanoma growth and increases survival of mice bearing melanoma [96]. Being overexpressed in metastatic melanoma, and particularly in targeted therapy-resistant clones, MC1R could represent a molecular target for metastatic melanoma and its inhibition a molecular strategy to delay resistance.

## Data Availability

Not applicable.

## Abbreviations

AC: Adenylyl Cyclase
ACTH: Adrenocorticotropic Hormone
Agrp: Agouti related protein
α-MSH: Alpha-melanocyte stimulating hormone
ATM: Ataxia Telangiectasia Mutated
BRAFi: BRAF inhibitors
Ca^2+^: Calcium
cAMP: Cyclic AMP
Dcs: Dendritic cells
DCT: Dopachrome Tautomerase
EPAC: Exchange Protein directly Activated by cAMP
ER: Endoplasmic reticulum
GoF: Gain of Function
GPCR: G-Protein Coupled Receptor
HSDD: Hypoactive Sexual Desire Disorders
IFN-γ: Interferon-gamma
MAPK: Mitogen-Activated Protein Kinase
MC1R: Melanocortin 1 receptor
MCRs: Melanocortin Receptors
MiTF: Microphthalmia inducing Transcription Factor
NER: Nucleotide Excision Repair
NfκB: Nuclear factor kappa-light-chain-enhancer of activated B cells
PI3K: Phosphatidylinositol 3-kinase
PIP2: Phosphatidylinositol-4,5-bisphosphate
PIP3: Phosphatidylinositol-3,4,5-triphosphate
PKA: Protein Kinase A
PLC: Phospholipase C
POMC: Pro-opiomelanocortin hormone
PPAR-γ: Peroxisome Proliferator-Activated Receptor-γ
PTEN: Phosphatase and TENsin homolog
RHC: Red-Hair-Colour
RTK: Tyrosine Kinase Receptor
TYR: Tyrosinase
TNF-α: Tumor Necrosis Factor alpha
TRP: Tyrosinase Related Protein
XPA: Xeroderma Pigmentosum complementation group A
